# Lack of Effectiveness of Repurposed Drugs for COVID-19 Treatment

**DOI:** 10.3389/fimmu.2021.635371

**Published:** 2021-03-11

**Authors:** Miguel Angel Martinez

**Affiliations:** IrsiCaixa, Hospital Universitari Germans Trias i Pujol, Universitat Autònoma de Barcelona, Badalona, Spain

**Keywords:** COVID-19, repurposed drugs, treatment efficacy, HIV, HCV

## Introduction

The magnitude of the coronavirus disease 2019 (COVID-19) pandemic caused by severe acute respiratory syndrome coronavirus 2 (SARS-CoV-2) has prompted the repurposing of several drugs to quickly stop the morbidity, mortality, and spread of this new disease. Repurposed drugs tested to fight COVID-19 have been chosen mainly on the basis of promising *in vitro* efficacy against SARS-CoV-2 or on previous therapeutic results with other human coronavirus diseases, such as severe acute respiratory syndrome (SARS) and Middle East respiratory syndrome (MERS) (1). Numerous clinical trials have already been completed, but no repurposed drug evaluated to date has been found that could significantly impact the course of COVID-19 pandemic (2).

Our experience with previous viral pandemics, such as human immunodeficiency virus type 1 (HIV-1) and hepatitis C virus (HCV), has taught us that precise design and target specificity will be essential to obtaining potent and successful antivirals against SARS-CoV-2.

## Failure of Repurposed Drugs

Repurposed drugs that have been explored more thoroughly since the beginning of the COVID-19 pandemic include remdesivir, favipiravir, lopinavir-ritonavir, ribavirin, interferon, and hydroxychloroquine (1). Favipiravir, a purine nucleoside analog broad-spectrum inhibitor of viral RNA-dependent RNA polymerase (RdRp), approved for treatment of influenza virus infection in Japan, was chosen due to its *in vitro* activity against SARS-CoV-2, nevertheless, there is no evidence of its clinical efficacy. A prospective, randomized, open-label trial of early vs. late favipiravir in hospitalized patients with COVID-19 has shown that favipiravir did not significantly improve viral clearance (3). Lopinavir-ritonavir, a HIV-1 protease inhibitor, was investigated due to its SARS-CoV antiviral activity in tissue culture and infected patients. However, the lopinavir-ritonavir combination exhibited no clinical benefit against SARS-CoV-2 (4). From the very beginning of the COVID-19 pandemic, remdesivir has been the most promising drug against SARS-CoV-2. This adenosine nucleotide analog prodrug, a potentially inhibitor of RdRp, was initially developed by Gilead Sciences to treat filoviruses, such as the Ebola virus, and was explored due to its broad-spectrum antiviral activity in tissue culture and animal models against filoviruses, paramyxoviruses, pneumoviruses, and pathogenic coronaviruses, including SARS-CoV and MERS-CoV. Randomized controlled trials (RCTs) have found no effect of remdesivir on mortality (5). This drug has been approved to treat COVID-19 in the USA and Europe, but conclusive results to support the use of remdesivir are lacking (6, 7).

The use of aminoquinoline drugs chloroquine and hydroxychloroquine is paradigmatic of the failure of repurposed drugs to treat COVID-19. These cheap drugs are generic antimalarials used to treat amoebic liver abscess and rheumatic disease. The early promising results with these two drugs showing antiviral activity against SARS-CoV-2 at micromolar concentrations in tissue culture and their clinical benefit in dubious observational trials of a few patients positioned them at the forefront of possible treatments for COVID-19. However, large observational clinical trials and RCTs have shown no effect of hydroxychloroquine in reducing mortality and/or mobility (2). Moreover, in some studies, a worse infection course was observed in hospitalized patients treated with these aminoquinolines (8). Importantly, recent studies have demonstrated that chloroquine does not inhibit infection of human lung cells with SARS-CoV-2 (9). Previous studies have shown that chloroquine and hydroxychloroquine inhibit the ability of SARS-CoV-2 to infect African green monkey kidney-derived Vero cells. However, when Vero cells were engineered to express TMPRSS2, a cellular protease that activates SARS-CoV-2 for entry into lung cells rendered SARS-CoV-2-infected Vero cells insensitive to chloroquine (9). Furthermore, chloroquine does not block infection with SARS-CoV-2 in TMPRSS2-expressing human lung Calu-3 cells, indicating that chloroquine targets a pathway for viral activation that is not active in lung cells and is unlikely to protect against the spread of SARS-CoV-2.

These results emphasize the necessity of being cautious with observed drug inhibition of viral replication in tissue culture. Ivermectin, another cheap antiparasitic drug with *in vitro* efficacy against SARS-CoV-2, is being prescribed as a preventative against COVID-19. However, the evidence that ivermectin protects people from COVID-19 is limited (10). We should be prudent using ivermectin, or other potential drugs, outside clinical trials. In some countries, ivermectin is being also administered to SARS-CoV-2 infected patients. Different doses and posology have been used and confounding results have been reported. A recent pilot clinical trial found no significant differences in detection of SARS-CoV-2 RNA from nasopharyngeal swabs at days four and seven after treating with a single oral dose of 400 mcrg/Kg of ivermectin (11). Virus target specificity (e.g., isolation or drug-resistant viruses) should be tested and demonstrated before initiating treatments in virus-infected patients. As hydroxychloroquine showed no effect in SARS-CoV-2 infection in non-human primates (12), testing animal models will be preferable before translating these drugs to humans.

## Hit Early, Hit Hard

In addition to drugs specifically aimed to inhibit SARS-CoV-2 replication, therapeutics that modulate inflammation have also been tested and, in this case, they seem to be a more effective therapeutic strategy for treating COVID-19 morbidity and mortality. Immunomodulators are being tested in several clinical trials for the treatment of SARS-CoV-2-generated cytokine storm. However, data to support the use of one of the most explored compounds to modulate inflammation, tocilizumab, a monoclonal antibody against interleukin-6 receptors, come largely from observational studies (13). Large RCTs with tocilizumab should provide answers regarding its clinical benefit.

Immunomodulators that appear to work are corticosteroids. A recent RTC performed with dexamethasone showed that, in patients with moderate or severe COVID-19, dexamethasone plus standard care significantly increases survival and reduces morbidity (14, 15). Other drugs that could offer clinical effects despite the lack of specific evidence for COVID-19 include anti-androgens, statins, N-acetyl cysteine, ACE2 inhibitors, angiotensin receptor blockers, and direct TMPRSS-2 inhibitors (16).

Although immunomodulators may be an excellent clinical tool, it is desirable to potently and specifically stop SARS-CoV-2 replication after the onset of the first COVID-19 symptoms to avoid the pathogenic course of the disease. Ideally, we should stop SARS-CoV-2 in the first days of the infection. For example, neuraminidase inhibitors may not produce any detectable effect in a patient hospitalized with severe influenza virus infection, but can be useful in preventing the development of severe disease. The most appropriate therapy goal of an acute viral infection is therefore not to cure severe disease, but to keep the disease from becoming severe, and prevent hospitalization. Treatment early in the course of illness could also limit person-to-person transmission.

A way to stop the early spread of SARS-CoV-2 will be through a sterilizing vaccine. SARS-CoV-2-neutralizing antibodies have been associated with protection (17). This is not surprising as natural infection induces both mucosal antibody responses (secretory IgA) and systemic antibody responses (IgG). The upper respiratory tract is mostly protected by secretory IgA, whereas the lower respiratory tract is mostly protected by IgG. Because most vaccines currently in development will be administered intramuscularly or intradermally, they will induce mostly IgG, but no secretory IgA (18). Therefore, these vaccines would probably prevent disease but not generate sterilizing immunity; that is, they may still allow for transmission of the virus (18). In this scenario, the current pandemic will require different strategies utilized in concert, including an effective vaccine and competent antivirals.

## HIV: A Success Story

HIV therapy is arguably among the most successful in treating any single human disease. The success of HIV therapeutics is illustrated by the number of antiretroviral agents and unique drug classes available (19). To date, the Food and Drug Administration (FDA) has approved 23 drugs to treat HIV infection. Based on their molecular mechanism and drug-resistance profile, antiretrovirals are classified into eight different classes: nucleoside reverse transcriptase inhibitors, non-nucleoside reverse transcriptase inhibitors, protease inhibitors, fusion inhibitors that block HIV entering CD4+ cells, CCR5 antagonists that block the CCR5 co-receptor that HIV needs to enter the cells, integrase inhibitors, attachment inhibitors that bind HIV glycoprotein 120, and post-attachment inhibitors that block cellular CD4 receptor. In addition to antiretrovirals, one pharmacokinetic enhancer has been approved to increase antiretroviral effectiveness. In contrast to the diffuse viral targets of most of the repurposed drugs mentioned above, antiretroviral target specificity was defined through the *in vitro* or *in vivo* selection of HIV-resistant variants for the different drugs. No antiretroviral has been approved in the absence of a specific viral target.

Although the virus was discovered in 1983, few antiretroviral treatment options existed for HIV infection before 1996. HIV therapeutics consisted mainly of prophylaxis against common opportunistic pathogens and managing AIDS-related illnesses. The development of HIV reverse transcriptase and protease inhibitors in the mid-1990s, and the introduction of drug regimens that combined these two classes of inhibitors to increase their efficacy, completely revolutionized the clinical approach to HIV. These combination treatments transformed HIV infection from a life-threatening disease to a manageable chronic disease. The success of antiretroviral therapies has strongly impacted the development of therapies against other viral infections. The best example is HCV, another pandemic, life-threatening, human viral infection discovered in 1989.

The first generation of FDA-approved HCV drugs included interferon alfacon-1 (approval year: 1997, discontinued in 2013 due to severe adverse events), ribavirin (1998), pegylated interferon alfa-2b (2001), and pegylated interferon alfa-2a (2002) (20). Although these drugs had low cure rates, a treatment duration of 48 weeks, and may cause severe adverse events, they were the only standard-of-care treatments over a decade. Interferons and ribavirin were chosen because they exhibited a certain inhibitory capacity against other viral infections, and their low effectivity is largely due to their low specificity against HCV. Fortunately, the development of direct-acting antivirals (DAAs) targeting the two main HCV enzymes, NS3 protease and RdRp (20), has decreased treatment duration to 8 weeks and increased the cure rate to nearly 100%. DAA therapy is among the best examples of success in the fight against viral infections. DAAs have transformed HCV management and opened the door to the global eradication of HCV.

## Discussion

Patients infected with HIV or HCV have a prolonged course of infection measured in months or years, during which they are asymptomatic or only mildly ill, providing ample opportunity to intervene with an antiviral drug. Because viremia is prolonged and relatively steady, a patient can serve as his own control to measure a drug effect. Although the situation is quite different for patients who have developed COVID-19, a rapidly progressive disease in whom might be more difficult to expect an antiviral to provide detectable benefit and more difficult to diagnose in its earlier stages when antiviral approaches would be more likely to be effective, HIV-1 and HCV examples should be mirrors in which we should look for antiviral solutions.

It can be argue that a repurposed drug that has not shown any benefit in hospitalized COVID-19 patients might still be useful in slowing the development of illness, preventing severe disease and making hospitalization unnecessary. In the absence of vaccines, a repurposed drug with limited antiviral activity might be given to persons who have been exposed to an infected individual, or are in a situation in which exposure is likely to occur (post- or pre-exposure prophylaxis). However, such benefit would be more difficult to demonstrate than a standard RCT, but could still be tested. A combinatorial approach of repurposing drugs targeting both the virus and host target mechanisms has been also proposed for the management of COVID-19 severity (21). The recent resolution of the crystal structures of the three most likely SARS-CoV-2 targetable proteins (spike, RdRp, and the main protease) is allowing the identification of first-generation SARS-CoV-2-specific antivirals (22–24). Even if the benefits of SARS-CoV-2-specific antivirals remain to be elucidated, we should quickly move these first-generation specific and potent antivirals to the clinic.

Antiviral drugs approved for the treatment of human virus infectious diseases have saved tens of millions of human beings over the last decades. It is a challenge to pursue effective, low-toxicity, and well-tolerated drugs that enhance patient compliance and drug administration. Nevertheless, effective antivirals will positively impact COVID-19 therapy, and SARS-CoV-2 transmission and eradication.

The success of antiretroviral and antiviral therapies developed against HIV and HCV should provide a point of reference for SARS-CoV-2 drug development and a roadmap for the development of novel COVOD-19 prevention strategies.

## Author Contributions

MM designed and wrote this manuscript.

## Conflict of Interest

The author declares that the research was conducted in the absence of any commercial or financial relationships that could be construed as a potential conflict of interest.
